# JAMI: fast computation of conditional mutual information for ceRNA network analysis

**DOI:** 10.1093/bioinformatics/bty221

**Published:** 2018-04-06

**Authors:** Andrea Hornakova, Markus List, Jilles Vreeken, Marcel H Schulz

**Affiliations:** 1 Max Planck Institute for Informatics, Saarland University, Saarland Informatics Campus, Saarbrücken, Germany; 2 Cluster of Excellence MMCI, Saarland University, Saarland Informatics Campus, Saarbrücken, Germany

## Abstract

**Motivation:**

Genome-wide measurements of paired miRNA and gene expression data have enabled the prediction of competing endogenous RNAs (ceRNAs). It has been shown that the sponge effect mediated by protein-coding as well as non-coding ceRNAs can play an important regulatory role in the cell in health and disease. Therefore, many computational methods for the computational identification of ceRNAs have been suggested. In particular, methods based on Conditional Mutual Information (CMI) have shown promising results. However, the currently available implementation is slow and cannot be used to perform computations on a large scale.

**Results:**

Here, we present JAMI, a Java tool that uses a non-parametric estimator for CMI values from gene and miRNA expression data. We show that JAMI speeds up the computation of ceRNA networks by a factor of ∼70 compared to currently available implementations. Further, JAMI supports multi-threading to make use of common multi-core architectures for further performance gain.

**Requirements:**

Java 8.

**Availability and implementation:**

JAMI is available as open-source software from https://github.com/SchulzLab/JAMI.

**Supplementary information:**

Supplementary data are available at *Bioinformatics* online.

## 1 Introduction

MicroRNAs (miRNAs) are ∼23 nt long RNAs that play an important role in the regulation of transcript abundance in mammalian cells. They are estimated to regulate at least half of the genes in the human genome (Friedman *et al.*, 2009) and thus affect important biological processes and show deregulation in many diseases (Jiang *et al.*, 2009). Several miRNAs often regulate the same transcript in a combinatorial fashion and many transcripts are regulated by the same miRNAs, leading to complex genome-wide networks of co-regulation (Tsang *et al.*, 2010). In these competing endogenous RNA (ceRNA) networks, ceRNA genes that carry binding sites for the same miRNA(s) compete over the limited pool of available miRNA molecules (Arvey *et al.*, 2010; Salmena *et al.*, 2011; Tay *et al.*, 2014). Several examples of ceRNA crosstalk have already been verified, including many genes involved in cancer such as *PTEN* (Poliseno *et al.*, 2010). This evidence has sparked interest in developing systematic methods for inferring ceRNA interactions from gene and miRNA expression data, reviewed in (Le *et al.*, 2017).

With the emergence of large-scale studies providing gene and miRNA expression data for hundreds of samples, it has become possible to infer ceRNA interactions computationally and several approaches have been suggested to achieve this. Sumazin et al. proposed the use of conditional mutual information in their method HERMES (Sumazin *et al.*, 2011), which was later implemented as part of the CUPID software package (CUPID step III) (Chiu *et al.*, 2015). While this method was applied successfully for inferring ceRNA networks for approximately 450 000 gene pairs (Chiu *et al.*, 2017), the current implementation is very slow and poses a bottleneck for the construction of large-scale networks.

This issue has motivated other researchers to design alternative approaches that are faster. For example methods based on linear correlation (Liu *et al.*, 2017; Paci *et al.*, 2014; Wang *et al.*, 2015). However, in contrast to CUPID, the linearity assumption limits the accuracy of these methods (Le *et al.*, 2017). We thus sought to speed up the computations of CMI values as the only known non-linear alternative for facilitating the efficient construction of large-scale ceRNA networks.

## 2 Results and discussion

**Fig. 1.**
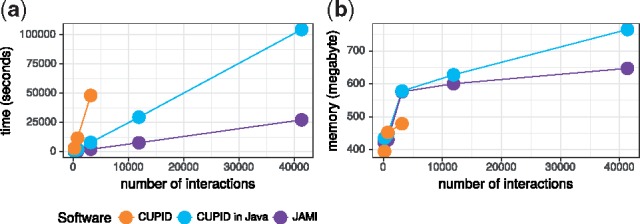
Performance comparison between JAMI, CUPID (Matlab) and CUPID (Java). (**a**) Process user time in seconds. (**b**) Peak memory usage

Here, we present JAMI, a novel implementation of the CMI computation step of CUPID (Chiu *et al.*, 2015). Like CUPID, JAMI uses adaptive partitioning for estimating CMI values (Darbellay and Vajda, 1999). This non-parametric estimator is consistent and makes no assumption on the distribution of the data and can thus be used with expression data from any technology. JAMI uses efficient data structures in Java to implement the three-dimensional data partitioning for the computation of CMI values. In contrast to CUPID, JAMI was carefully designed to support multi-threading (Supplementary Fig. S1). In Figure 1, we show that JAMI achieves a substantially better single-threaded runtime compared to CUPID implemented in either Matlab or Java. For the latter comparison, we carefully re-implemented the original CUPID method in Java.

Both JAMI and CUPID rank expression values before the CMI computation. In CUPID, all expression values of 0 are assigned different ranks. This introduces bias and results in positive CMI values even if genes are not expressed in any sample. To avoid this, we extended JAMI to be zero expression aware, and demonstrate that this has considerable effect on the results (Supplementary Figs S2–S5).

Preparing the input for CUPID is tedious and requires separate expression and miRNA interaction files as input for every gene pair of interest. In contrast, JAMI accepts two expression matrices as input, one for gene and one for miRNA expression, and filters these automatically for the data needed. In addition, JAMI offers great flexibility with regards to defining the triplets of interest, making it much more convenient to use JAMI in settings where several genes are of interest. JAMI output files can be directly imported in network analysis tools such as Cytoscape (Shannon *et al.*, 2003). Moreover, JAMI does not require an expensive Matlab^®^ license like CUPID, making it available to a broader audience. To make sure that JAMI can also be used conveniently in a scripting language, we implemented the RJAMI wrapper package for R (http://github.com/SchulzLab/RJAMI).

We illustrate the potential of JAMI by constructing a ceRNA interaction network from the TCGA breast cancer data set (TCGA, 2012) for known ceRNAs (Tay *et al.*, 2014) (Supplementary Fig. S6, see user manual for a step by step guide). The resulting network appears to be much denser than what is reported in the literature, emphasizing the importance of robust tools for ceRNA network inference from widely available expression data.

An open question in the field is whether linear or non-linear methods are better suited for ceRNA network inference (Le *et al.*, 2017). Answering this question was thus far impeded by the lack of a fast tool for computing CMI values. JAMI overcomes this research barrier and facilitates comparisons with correlation-based method such as sensitivity correlation (Paci *et al.*, 2014) (Supplementary Fig. S7).

In conclusion, JAMI is a fast, freely available and well-documented (http://jami.readthedocs.io/) tool primarily targeted at the inference of ceRNA networks. However, its implementation is general and may be used to study other modulators of gene–gene interactions, e.g. transcription factors (Flores *et al.*, 2013).

## Supplementary Material

Supplementary Data

## References

[bty221-B1] Arvey A. et al (2010) Target mRNA abundance dilutes microRNA and siRNA activity. Mol. Syst. Biol., 6, 363. 20404830 10.1038/msb.2010.24 PMC2872614

[bty221-B2] Chiu H.S. et al (2015) Cupid: simultaneous reconstruction of microRNA-target and ceRNA networks. Genome Research, 25, 257–267. 25378249 10.1101/gr.178194.114 PMC4315299

[bty221-B3] Chiu H.-S. et al (2017) High-throughput validation of ceRNA regulatory network. BMC Genomics, 18, 418. 28558729 10.1186/s12864-017-3790-7 PMC5450082

[bty221-B4] Darbellay G.A., Vajda I. (1999) Estimation of the information by an adaptive partitioning of the observation space. IEEE Trans. Information Theory, 45, 1315–1321.

[bty221-B5] Flores M. et al (2013) Gene regulation, modulation, and their applications in gene expression data analysis. Adv. Bioinformatics, 2013, 1. 10.1155/2013/360678 PMC3610383 23573084

[bty221-B6] Friedman R.C. et al (2009) Most mammalian mRNAs are conserved targets of microRNAs. Genome Res., 19, 92–105. 18955434 10.1101/gr.082701.108 PMC2612969

[bty221-B7] Jiang Q. et al (2009) miR2Disease: a manually curated database for microRNA deregulation in human disease. Nucleic Acids Res., 37, D98–D104. 18927107 10.1093/nar/gkn714 PMC2686559

[bty221-B8] Le T.D. et al (2017) Computational methods for identifying miRNA sponge interactions. Brief. Bioinformatics. 18, 577–590. 27273287 10.1093/bib/bbw042

[bty221-B9] Liu C. et al (2017) Cancer-Related Triplets of mRNA-lncRNA-miRNA Revealed by Integrative Network in Uterine Corpus Endometrial Carcinoma. BioMed Res. Int., 2017, 3859582. 28280730 10.1155/2017/3859582 PMC5320387

[bty221-B10] Paci P. et al (2014) Computational analysis identifies a sponge interaction network between long non-coding RNAs and messenger RNAs in human breast cancer. BMC Syst. Biol., 8, 83. 25033876 10.1186/1752-0509-8-83 PMC4113672

[bty221-B11] Poliseno L. et al (2010) A coding-independent function of gene and pseudogene mRNAs regulates tumour biology. Nature, 465, 1033–1038. 20577206 10.1038/nature09144 PMC3206313

[bty221-B12] Salmena L. et al (2011) A ceRNA hypothesis: the Rosetta Stone of a hidden RNA language? Cell, 146, 353–358. 21802130 10.1016/j.cell.2011.07.014 PMC3235919

[bty221-B13] Shannon P. et al (2003) Cytoscape: a software environment for integrated models of biomolecular interaction networks. Genome Res., 13, 2498–2504. 14597658 10.1101/gr.1239303 PMC403769

[bty221-B14] Sumazin P. et al (2011) An extensive MicroRNA-mediated network of RNA-RNA interactions regulates established oncogenic pathways in glioblastoma. Cell, 147, 370–381. 22000015 10.1016/j.cell.2011.09.041 PMC3214599

[bty221-B15] Tay Y. et al (2014) The multilayered complexity of ceRNA crosstalk and competition. Nature, 505, 344. 24429633 10.1038/nature12986 PMC4113481

[bty221-B16] TCGA, T. C. G. A. C. (2012) Comprehensive molecular portraits of human breast tumours. Nature, 490, 61–70. 23000897 10.1038/nature11412 PMC3465532

[bty221-B17] Tsang J.S. et al (2010) Genome-wide dissection of microRNA functions and co-targeting networks using gene-set signatures. Mol. Cell, 38, 140–153. 20385095 10.1016/j.molcel.2010.03.007 PMC3110938

[bty221-B18] Wang P. et al (2015) Identification of lncRNA-associated competing triplets reveals global patterns and prognostic markers for cancer. Nucleic Acids Res., 43, 3478–3489. 25800746 10.1093/nar/gkv233 PMC4402541

