# Supplementary material for: JAMI: fast computation of conditional mutual information for ceRNA network analysis
Source: Bioinformatics. 2018 Apr 6;34(17):3050–1. doi: 10.1093/bioinformatics/bty221 (PMC6129307; doi:10.1093/bioinformatics/bty221)
Supplement: Supplementary Data [file bty221_supplemental_material.pdf]

JAMI - Fast computation of Conditional Mutual Information for ceRNA network analysis

Supplemental Material

Andrea Hornakova[1,†], Markus List[1,†], Jilles Vreeken[1,2] and Marcel H. Schulz [1,2]

[1]Max Planck Institute for Informatics, Saarland Informatics Campus, 66123 Saarbrücken, Germany
[2]Cluster of Excellence MMCI, Saarland University, Saarland Informatics Campus, 66123 Saarbrücken, Germany

†joint first authors

Contents

Usage notes:

Implementation improvements:

Benchmark dataset:

Profiling of CPU time and memory usage

Parallelization:

Dealing with zero expression values:

Comparison to correlation-based Methods

References

Usage notes:

JAMI comes with a detailed user manual located at http://jami.readthedocs.io. The manual provides usage instructions as well as a step-by-step guide to help users produce ceRNA interaction networks. The user is also shown how to load them into 3rd party software like Cytoscape for downstream analysis and visualization.

Alternatively, we also implemented a wrapper package for JAMI in R. Installation details and a detailed user guide are shown at http://github.com/SchulzLab/RJAMI and in the package vignette.

Implementation improvements:

In addition to parallelizing the execution of the CMI computation, we made several improvements that contribute to making JAMI considerably faster even in single thread mode. These include:

- In contrast to CUPID where gene and miRNA expression data have to be read from file for each gene pair, JAMI reads required expression data for all considered gene pairs at once and keeps it in memory for faster access.
- For a specific dataset, the initial cube used for iterative partitioning will always be the same. We use a single instance of this cube and pass it by reference.
- After performing a split of a cube in iterative partitioning, CUPID requires O(n^2) for assigning the points to the new cubes, whereas JAMI requires O(n).
- To compute CMI values, CUPID loops through all points and retrieves their respective coordinates in three dimension by lookups in three indices. In contrast, JAMI follows the points along one coordinate and performs index lookups for only two dimensions.
- For the random permutations, we do not create all data structures from scratch but reuse them efficiently, while permuting the indices.

Benchmark dataset:

The benchmark dataset was generated using paired miRNA and gene RNA-seq data from The Cancer Genome Project. Benchmark genes were selected using partial correlation to identify promising candidate genes with a large number of interactions. Genes in this network have been sorted by their network node degree and the top 5, 10, 20, 40, or 80 genes were selected for the benchmark. Putative gene-miRNA interactions have been selected based on the output of an cross-validated elastic net regression with alpha = 0.5 (balancing ridge and lasso regression). Here, the response variable is the gene expression vector and the miRNA expression vectors serve as regressors. Only miRNAs with an coefficient < -0.05 have been selected to ensure a meaningful negative regulation of the miRNA is given. A custom R script was used to infer shared miRNAs between pairs of genes and to generate the necessary input files for CUPID and JAMI.

Profiling of CPU time and memory usage

We used the linux tool GNU time (v1.7) to profile the average memory usage and CPU (user) time of:

1. As a baseline for our comparison, we executed CUPID with 182, 752, 3,142, 11,8765 and 41,338 interactions between 5, 10, 20, 40 and 80 genes, respectively. We could not produce timings for 40 and 80 gene cases due to excessive runtime (terminated after 48 hours).
2. We compared this against running JAMI with the -rankzeros flag (to ensure a fair comparison, since this step is missing in CUPID).
3. Since we expect that switching to a Java implementation already led to a tremendous improvement of the runtime, we also included an exact re-implementation of CUPID in JAMI. This mode can be used by setting the '-method cupid' flag when executing JAMI. Importantly, the difference between the runtime of 3. and 2. can be attributed to improvements in the implementation.

Parallelization:

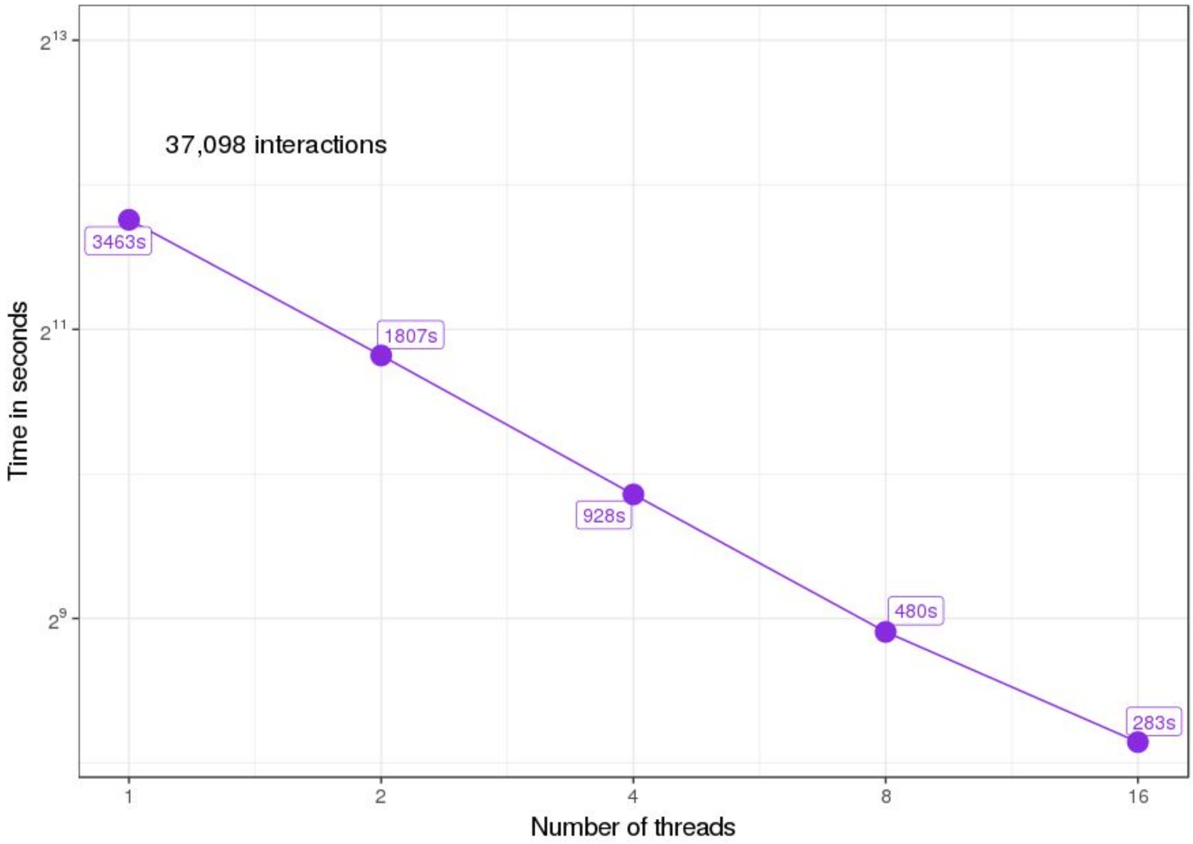

Supplemental Figure 1: JAMI executed on a test set of 37,098 interactions with varying number of threads illustrating a near-linear performance increase.

Dealing with zero expression values:

Before conditional mutual information can be computed, JAMI and CUPID transform the real valued input expression values into ranks. Due to this, the algorithm does not handle duplicate values correctly. While duplicates are typically not expected in gene and miRNA expression data, there is one exception. If no expression is measured in a sample (e.g. no reads have been mapped to a gene in next-generation sequencing data), the expression value will be zero. For lowly expressed genes, these zero expression values can make up a sizable fraction of the expression data, thus introducing a considerable bias into the CMI computation.

To address this issue, we extended the CMI algorithm in JAMI to handle zero expression values explicitly. More precisely, we introduce a preprocessing step in which zero expression values are split off the initial cube into a series of subcubes in which the corresponding dimension containing zero expression values is collapsed. The resulting square keeps a memory of the zero values that have been collapsed on the third axis in a HashSet to facilitate correct computation. If zero expression values are found on a second dimension this will also be collapsed, resulting in a line. If zero

expression values are found on the third axis those samples will be split off and result in a single bin that is not split further. Squares and lines are processed similar to cubes in that they are split at the center until they are balanced according to the chi squared test.

In this way, we avoid splitting intervals of zero expression values and assigning arbitrary ranks to these values. This allows us to compute accurate CMI values under the assumption that no other values are duplicated. To identify zero expression values, we check if the minimal expression value is duplicated. Thus, we can also accommodate log2 scaled data in which a pseudocount is added to obtain a large negative value for the zero expression case. For the ceRNA network introduced above, we show that considering zero expression values has a considerable impact on the results:

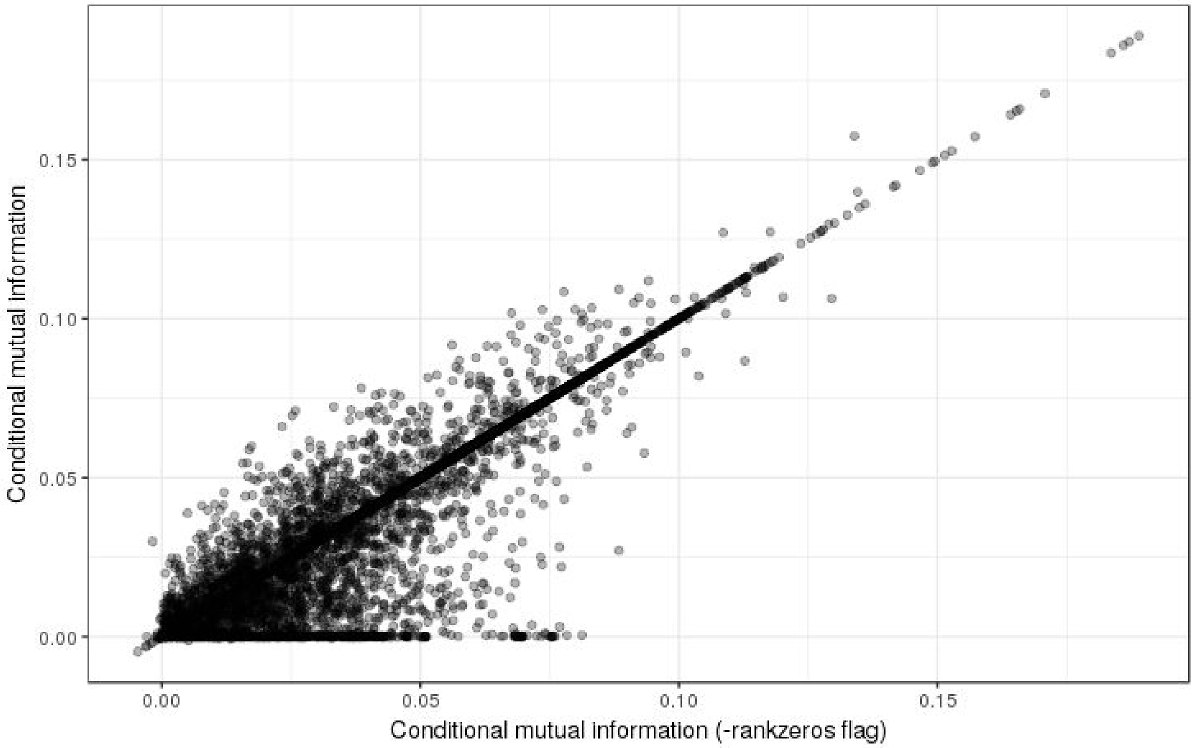

Supplemental Figure 2: Comparison of CMI values when accounting for duplicated zero expression values to the -rankzeros mode in which they are ignored. It is evident that the CMI values are affected by this. Notably, the -nozero mode produces positive CMI values even when a gene or miRNA is not expressed.

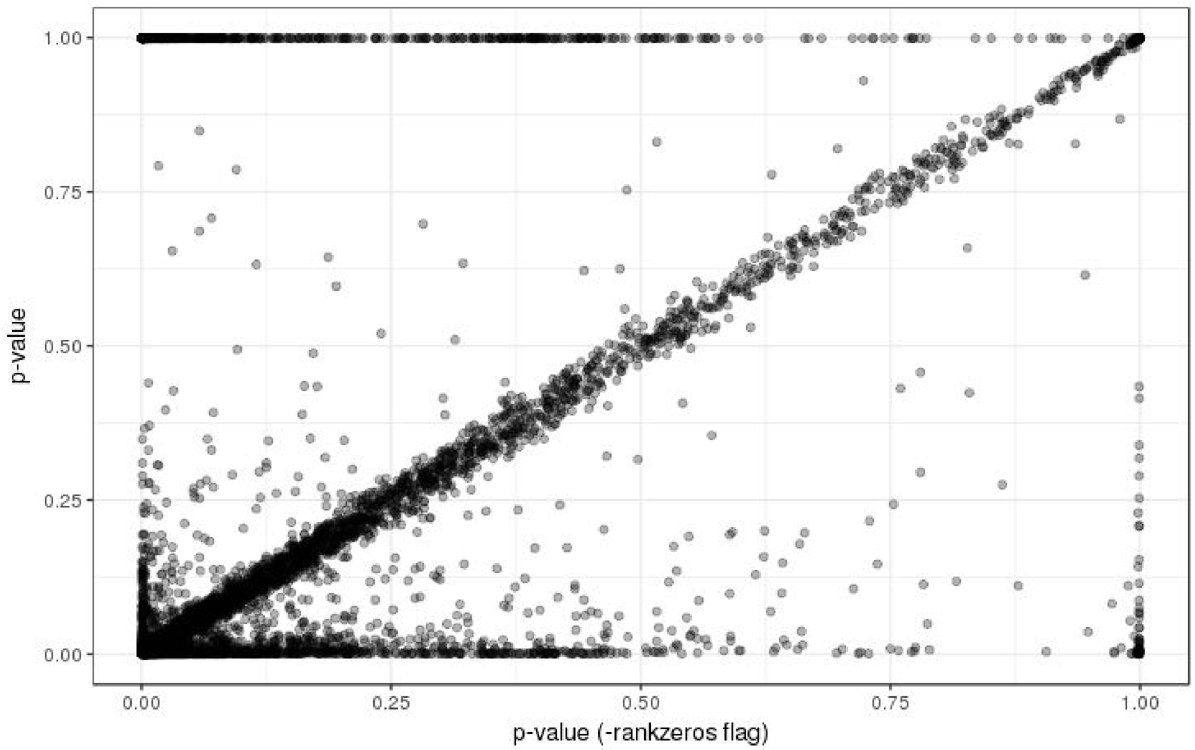

Supplemental Figure 3: Comparison of p-values when accounting for duplicated zero expression values to the -nozero mode in which they are ignored. As can be expected from the differences in CMI values shown in Supplemental Figure 2, p-values may change drastically when zero expression values are present.

Accounting for the bias introduced by duplicated zero expression value improves the results of the ceRNA interaction network we computed above. To test this, we iterated through each modulator gene and computed the ranks (highest CMI with rank = 1) of its interaction when accounting for zeros and when not accounting for zeros (-rankzeros mode). Next, we identified miRNA interactions with experimental evidence using miRTarBase [Chou2015] (version 7, downloaded December 6, 2017). We hypothesize that accounting for zeros improves the ranks of miRNA interactions with experimental evidence. Indeed, for the ceRNA network of Tay et al. [Tay2014], we observe a significant improvement in the rank (wilcoxon test p = 0.049 two-sided, 0.025 one-sided).

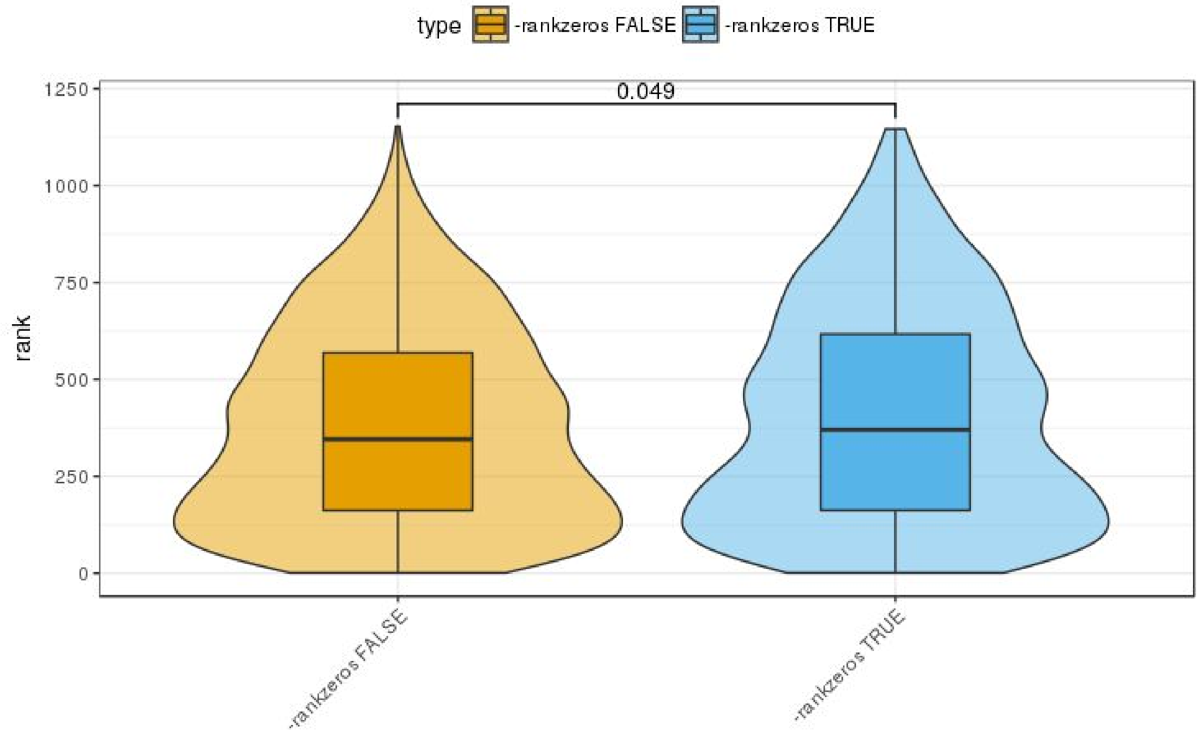

Supplemental Figure 4: Significant rank improvement for ceRNA interactions that involve miRNAs targeting the modulator gene and for which experimental evidence was reported in miRTarBase 7. Note that with the -nozero flag set JAMI will behave like CUPID and rank all values irrespective of the occurrence of zero expression values.

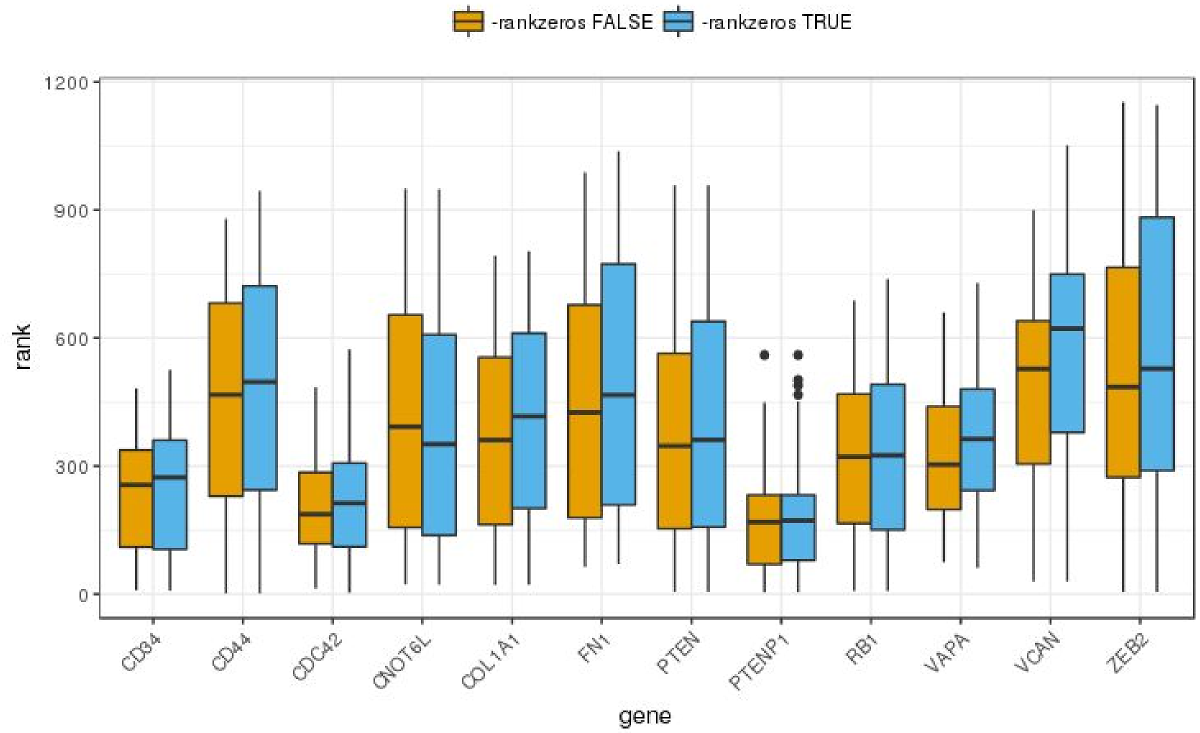

Supplemental Figure 5: Ranks of miRNAs for which experimental evidence was reported in miRTarBase 7, here shown for individual modulator genes. Note that with the -nozero flag set JAMI will behave like CUPID and rank all values irrespective of the occurence of zero expression values.

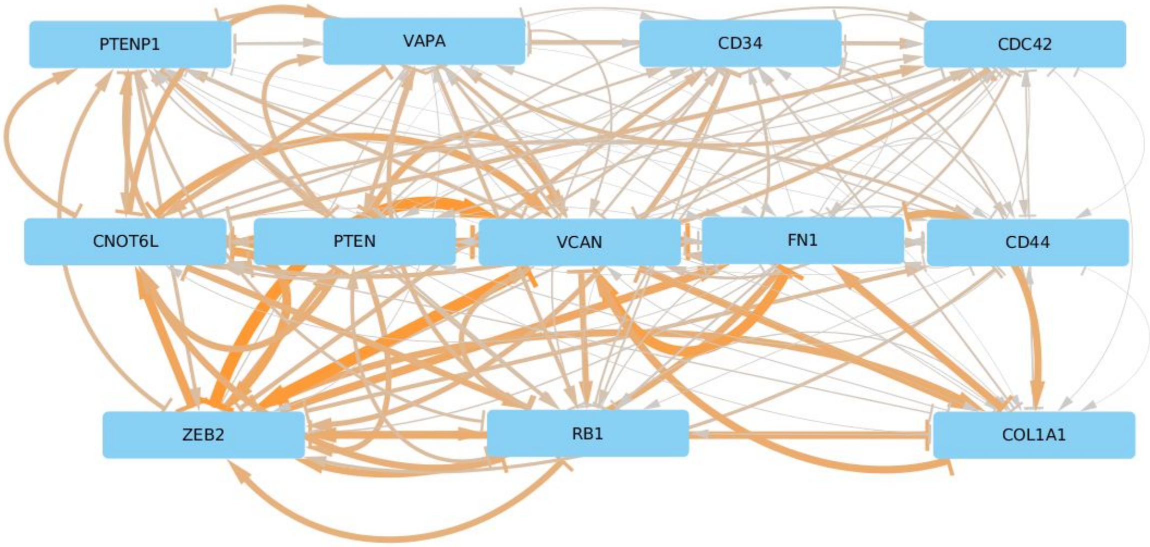

Supplemental Figure 6: JAMI inferred ceRNA network for known ceRNAs reported in Tay et al. 2014.

Comparison to correlation-based Methods

JAMI facilitates comparisons to correlation-based methods such as sensitivity correlation. The following plot shows that CMI and sensitivity correlation values have a moderate correlation of 0.67 when applied to our benchmark dataset.

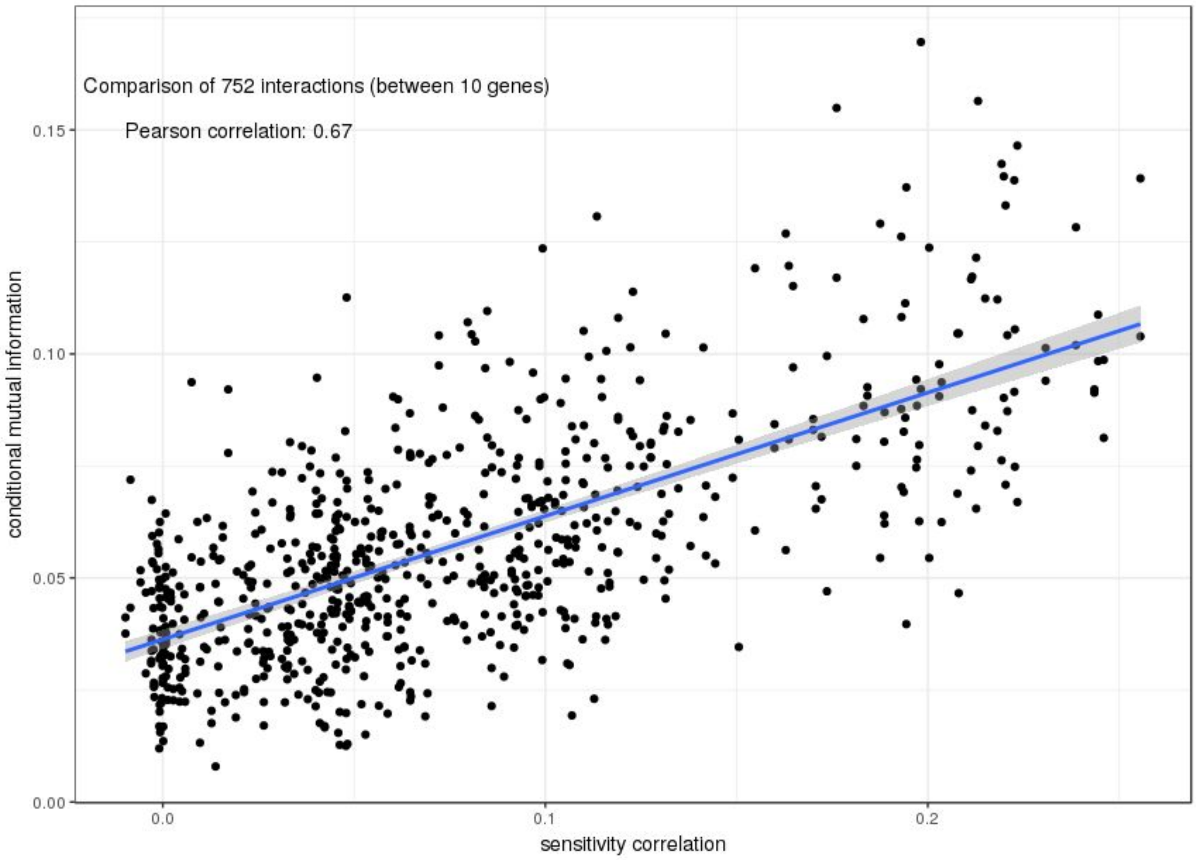

Supplemental Figure 7: Comparison between sensitivity correlation and conditional mutual information for a set of 752 matched interactions.

References

[Tay2014] Tay, Yvonne, John Rinn, and Pier Paolo Pandolfi. "The multilayered complexity of ceRNA crosstalk and competition." Nature 505, no. 7483 (2014): 344-352.

[Chou2015] Chou, Chih-Hung, Nai-Wen Chang, Sirjana Shrestha, Sheng-Da Hsu, Yu-Ling Lin, Wei-Hsiang Lee, Chi-Dung Yang et al. "miRTarBase 2016: updates to the experimentally validated miRNA-target interactions database." Nucleic acids research 44, no. D1 (2015): D239-D247.
